# Impact of the COVID-19 Pandemic on Health Care Utilization in the Vaccine Safety Datalink: Retrospective Cohort Study

**DOI:** 10.2196/48159

**Published:** 2024-01-23

**Authors:** Lei Qian, Lina S Sy, Vennis Hong, Sungching C Glenn, Denison S Ryan, Jennifer C Nelson, Simon J Hambidge, Bradley Crane, Ousseny Zerbo, Malini B DeSilva, Jason M Glanz, James G Donahue, Elizabeth Liles, Jonathan Duffy, Stanley Xu

**Affiliations:** 1 Research and Evaluation Kaiser Permanente Southern California Pasadena, CA United States; 2 Kaiser Permanente Washington Health Research Institute Seattle, WA United States; 3 Denver Health Ambulatory Care Services Denver, CO United States; 4 Center for Health Research Kaiser Permanente Northwest Portland, OR United States; 5 Kaiser Permanente Vaccine Study Center Kaiser Permanente Northern California Oakland, CA United States; 6 HealthPartners Institute Bloomington, MN United States; 7 Institute for Health Research Kaiser Permanente Colorado Aurora, CO United States; 8 Marshfield Clinic Research Institute Marshfield, WI United States; 9 Centers for Disease Control and Prevention Atlanta, GA United States; 10 Department of Health Systems Science Kaiser Permanente Bernard J. Tyson School of Medicine Pasadena, CA United States

**Keywords:** COVID-19 pandemic, health care utilization, telehealth, inpatient, emergency department, outpatient, vaccine safety, electronic health record, resource allocation, difference-in-difference, interrupted time series analysis

## Abstract

**Background:**

Understanding the long-term impact of the COVID-19 pandemic on health care utilization is important to health care organizations and policy makers for strategic planning, as well as to researchers when designing studies that use observational electronic health record data during the pandemic period.

**Objective:**

This study aimed to evaluate the changes in health care utilization across all care settings among a large, diverse, and insured population in the United States during the COVID-19 pandemic.

**Methods:**

We conducted a retrospective cohort study within 8 health care organizations participating in the Vaccine Safety Datalink Project using electronic health record data from members of all ages from January 1, 2017, to December 31, 2021. The visit rates per person-year were calculated monthly during the study period for 4 health care settings combined as well as by inpatient, emergency department (ED), outpatient, and telehealth settings, both among all members and members without COVID-19. Difference-in-difference analysis and interrupted time series analysis were performed to assess the changes in visit rates from the prepandemic period (January 2017 to February 2020) to the early pandemic period (April-December 2020) and the later pandemic period (July-December 2021), respectively. An exploratory analysis was also conducted to assess trends through June 2023 at one of the largest sites, Kaiser Permanente Southern California.

**Results:**

The study included more than 11 million members from 2017 to 2021. Compared with the prepandemic period, we found reductions in visit rates during the early pandemic period for all in-person care settings. During the later pandemic period, overall use reached 8.36 visits per person-year, exceeding the prepandemic level of 7.49 visits per person-year in 2019 (adjusted percent change 5.1%, 95% CI 0.6%-9.9%); inpatient and ED visits returned to prepandemic levels among all members, although they remained low at 0.095 and 0.241 visits per person-year, indicating a 7.5% and 8% decrease compared to pre-pandemic levels among members without COVID-19, respectively. Telehealth visits, which were approximately 42% of the volume of outpatient visits during the later pandemic period, were increased by 97.5% (95% CI 86.0%-109.7%) from 0.865 visits per person-year in 2019 to 2.35 visits per person-year in the later pandemic period. The trends in Kaiser Permanente Southern California were similar to those of the entire study population. Visit rates from January 2022 to June 2023 were stable and appeared to be a continuation of the use levels observed at the end of 2021.

**Conclusions:**

Telehealth services became a mainstay of the health care system during the late COVID-19 pandemic period. Inpatient and ED visits returned to prepandemic levels, although they remained low among members without evidence of COVID-19. Our findings provide valuable information for strategic resource allocation for postpandemic patient care and for designing observational studies involving the pandemic period.

## Introduction

The COVID-19 pandemic, which began in the United States in March 2020, resulted in sudden reductions in health care utilization due to factors including fear of exposure to the novel coronavirus (SARS-CoV-2), stay-at-home orders, and restriction of in-person health care delivery [1,2]. Multiple studies reported an abrupt decrease in in-person health care including inpatient visits, emergency department (ED) visits, outpatient visits, and a large increase in telehealth visits after the start of the pandemic [3-6]. However, these changes in health care utilization were assessed mostly during the early pandemic period in 2020.

Studies assessing the longer-term impact of the COVID-19 pandemic on health care utilization are limited. Mafi et al [7] evaluated trends in US ambulatory care patterns during the COVID-19 pandemic between March 2020 and February 2021. Another study led by Zachrison et al [8] evaluated changes in telehealth and inpatient health care utilization as of April 2021. Both studies were conducted before COVID-19–related restrictions were relaxed. For example, in California, effective as of June 15, 2021, Executive Order N-07-21 removed most restrictions including physical distancing because more than half of eligible Californians had received the full series of COVID-19 vaccinations [9]. A more recent study assessed health care utilization during the first 2 years of the COVID-19 pandemic, but it was conducted in Israel and limited to only primary care visits [10]. Thus, it remains unclear whether health care utilization returned to prepandemic levels across visit types by the end of 2021 in the United States.

Understanding the impact of the COVID-19 pandemic on health care utilization is important to health care organizations and policy makers for strategic planning of care services and resource allocation [6], as well as to investigators designing studies that plan to use observational health care data during the pandemic period [11,12]. This study aims to evaluate the changes in health care utilization across inpatient, ED, outpatient, and telehealth care settings during the COVID-19 pandemic among members enrolled in 8 geographically diverse integrated health care organizations across the United States from 2017 to 2021, including the period after the relaxation of COVID-19 restrictions. Since COVID-19–related care might have affected the secular patterns of health care utilization and potentially competed for resources allocated to non–COVID-19–related medical needs [13,14], the analyses were conducted separately among all members and members without COVID-19. We also conducted an exploratory analysis to assess trends through June 2023 at one of the largest sites (Kaiser Permanente Southern California [KPSC]).

## Methods

### Study Setting

We conducted a retrospective cohort study within 8 health care organizations (Kaiser Permanente: Southern California, Northern California, Northwest, Washington, and Colorado; HealthPartners; Denver Health; and Marshfield Clinic) participating in the Vaccine Safety Datalink (VSD) Project [15]. The VSD is a collaborative project between the Centers for Disease Control and Prevention’s Immunization Safety Office and integrated health care organizations, with over 9 million individuals annually enrollment representing approximately 3% of the US population. Each participating site creates standardized data sets with information on members’ demographic characteristics, membership enrollment, vaccination history, and medical encounters in the inpatient, ED, outpatient, and telehealth settings.

### Ethical Considerations

The institutional review board at each study site approved the study with a waiver of informed consent, given that the data-only retrospective research activities were minimal risk. To protect the privacy and confidentiality of human participants, all staff working on the research study were trained in procedures to protect the privacy of medical record information. Study participants were not compensated given the observational nature of the study.

### Study Population

The study population consisted of individuals of all ages who were active health plan members at any time from January 1, 2017, to December 31, 2021 from 8 participating VSD sites. Members who did not test positive for COVID-19 or have a medically attended COVID-19 diagnosis at any time during the study period were classified as “members without COVID-19.” The exploratory analysis to assess the more recent trends included KPSC members from January 1, 2022, to June 30, 2023.

### Health Care Utilization

Four visit types were evaluated based on care setting: inpatient visit, ED visit, outpatient visit, and telehealth visit. Telehealth visits were conducted synchronously using telephone or live video-audio interaction. The visit rates per person-year from these 4 care settings were calculated monthly during the study period from 2017 to 2021. The numerator was the visit count of each type, and the denominator was person-years of membership during a given month. We also calculated the overall visit rates (all 4 settings combined), the rates of combined outpatient and telehealth visits (as total ambulatory care), and the rates of combined in-person (inpatient, ED, and outpatient) visits.

### Statistical Analysis

#### Overview

Demographic characteristics (age, sex, and race or ethnicity) of the study population were described by year. Monthly visit rates overall and by visit type were plotted over time before and during the COVID-19 pandemic to examine trends.

We selected April-December 2020 and July-December 2021 as the early and later pandemic periods, respectively, to assess the short- and longer-term impact of the pandemic on health care utilization. The July-December 2021 period was selected as the later pandemic period because COVID-19–related restrictions were relaxed during this period.

#### Short-Term Impact Assessment

To assess the short-term impact of the pandemic on health care utilization during the early pandemic period, due to data volatility during this period, we performed difference-in-difference (DiD) analyses by comparing visit rates in each of the 3 periods of the early pandemic (April-June, July-September, and October-December) in 2020 to the January-February 2020 before the pandemic period; visit rates in the 4 corresponding periods in 2017-2019 were also calculated and used to control for seasonality (Figure S1A in Multimedia Appendix 1) [16]. We excluded March 2020 from the analysis because the COVID-19 pandemic was declared a national emergency on March 13, 2020. We estimated the ratio of rate ratios (RRRs) and 95% CIs by fitting Poisson regression models with the visit count as the dependent variable and the natural logarithm of person-years of membership as an offset, adjusting for overdispersion of the count data. The percent change of visit rates during the early pandemic period from the prepandemic period was calculated as (RRR – 1) × 100. Separate analyses were conducted for all visit types combined and by visit type.

#### Long-Term Impact Assessment

To assess the longer-term impact of the pandemic on health care utilization during the later pandemic period, interrupted time series (ITS) analyses were conducted with adjustment for prepandemic secular trends and seasonality by including calendar year and month in the model (Figure S1B in Multimedia Appendix 1) [17]. ITS analysis is a statistical method used to evaluate the impact of an intervention or event, which is introduced at a specific time point on time series data. A model with a linear function of the year was selected based on visual exploration of the trend plots and comparison with models with a quadratic form. Poisson regression models were used to estimate adjusted rate ratios (RRs) and 95% CI, comparing visit rates in the later pandemic period with the prepandemic period. The percent change in visit rates was calculated as (RR – 1) × 100. ITS analyses were conducted for all visits combined and separately by visit type.

COVID-19–related care associated with each wave of COVID-19 infection might have affected the secular patterns of health care utilization. We also conducted sensitivity analyses of health care utilization among members without evidence of COVID-19. For the exploratory analysis, we plotted monthly visit rates overall and by visit type. We also conducted ITS analysis to estimate the adjusted percent change of visit rates in July-December 2022 compared to prepandemic levels. All analyses were conducted using SAS (version 9.4; SAS Institute Inc).

## Results

Annual enrollment did not significantly change between 2017 and 2021, with the total number of enrolled person-years ranging from 10.9 to 11.5 million across the 8 VSD sites. Demographic characteristics of members enrolled in VSD sites remained relatively stable during 2017-2021 (Table 1).

**Figure 1:**
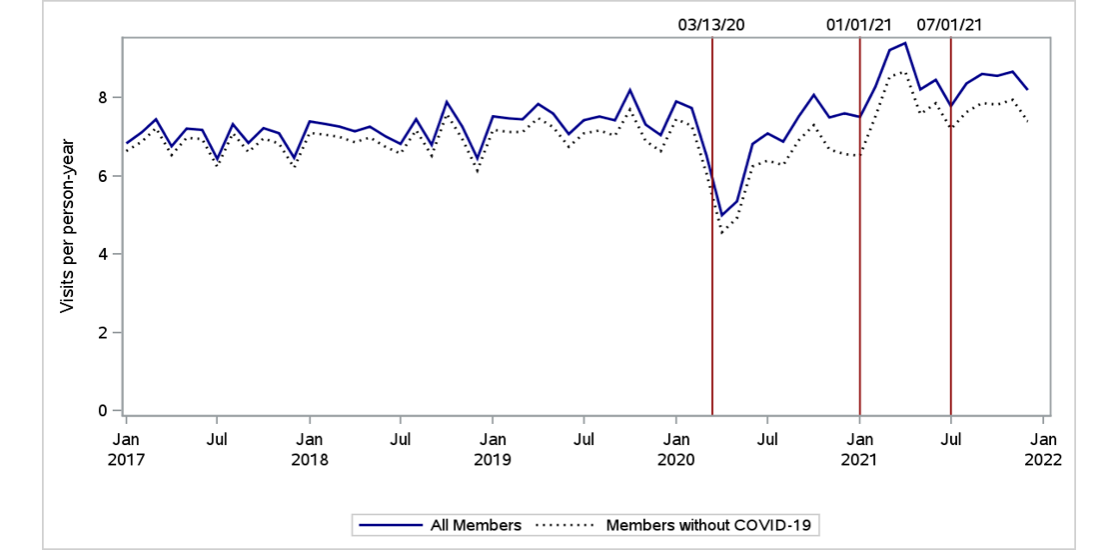
Monthly overall visit rate among all members and members without COVID-19 during 2017-2021. March 13, 2020: the COVID-19 pandemic was declared a national emergency; April-December 2020: early pandemic period; July-December 2021: later pandemic period.

**Figure 2:**
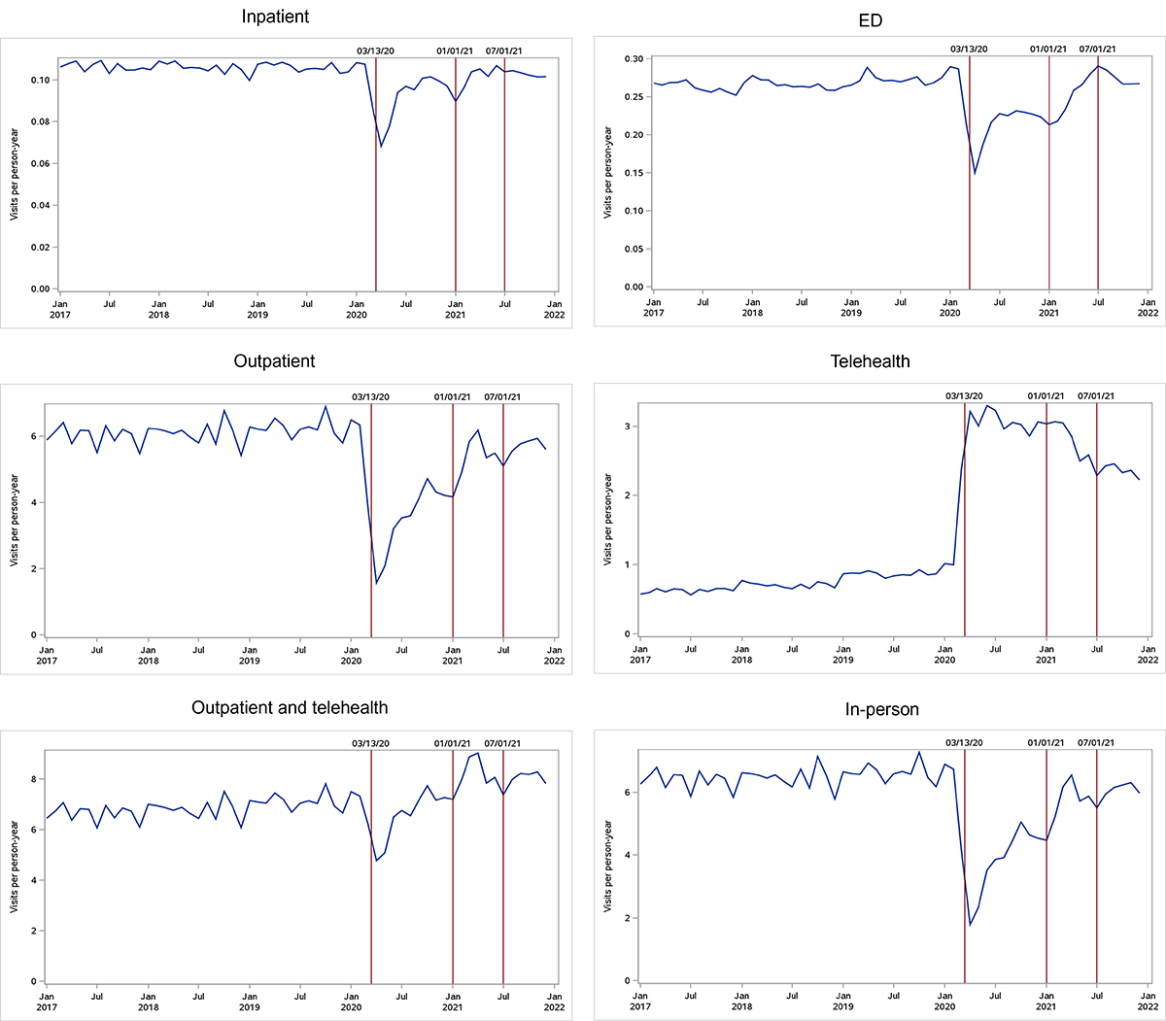
Monthly visit rate by care setting among all members during 2017-2021. March 13, 2020: the COVID-19 pandemic was declared as a national emergency; April-December 2020: early pandemic period; July-December 2021: later pandemic period. ED: emergency department.

The trend plot showed that the overall monthly visit rates fluctuated normally before March 2020, then decreased abruptly in March, reaching the lowest levels in April 2020, and then gradually increased afterward (Figure 1). The overall visit rates returned to prepandemic levels during 2021 and were stable after July 2021. Use trends differed by visit type (Figure 2). All in-person (inpatient, ED, and outpatient) visits decreased markedly in the beginning of the pandemic, and then gradually increased. Among the 3 in-person visit types, outpatient visits had the largest decrease at the beginning of the pandemic. Telehealth visits increased sharply after the start of the pandemic, and then slowly decreased, although they remained above the prepandemic levels. Combined outpatient and telehealth visit rates decreased in the early pandemic period and exceeded prepandemic levels in 2021.

The overall visit rate decreased from 7.82 visits per person-year in January-February 2020 to 5.72 visits per person-year in April-June 2020, a 26.4% reduction (95% CI –36.3% to –15.1%); it returned to prepandemic levels in October-December 2020 (adjusted percent change –0.4%, 95% CI –6.3% to 5.9%; Table 2). The overall visit rate increased by 5.1% (95% CI 0.6%-9.9%) to 8.36 visits per person-year in July-December 2021 compared with the same months in the prepandemic period (Table 3). The rates of inpatient, ED, and outpatient visits decreased by 24.9% (95% CI –33.3% to –15.3%), 35.5% (95% CI –43.7% to –26.2%), and 64.2% (95% CI –71.9% to –54.3%) in April-June 2020 and by 5.3% (95% CI –8.3% to –2.2%), 19.2% (95% CI –22.7% to –15.5%), and 30.5% (95% CI –34.4% to –26.4%) in October-December 2020 compared to the rates of 0.108, 0.288, and 6.42 visits per person-year in January-February 2020, respectively (Table 2). While the rates of inpatient and ED visits returned to prepandemic levels in July-December 2021, the rate of outpatient visits was still 12.5% lower (95% CI –16.8% to –8.0%; Table 3). Telehealth visit rates increased by 218.3% (95% CI 186.4%-253.6%) from 1.01 visits per person-year in January-February 2020 to 3.17 visits per person-year in April-June 2020 (Table 2). With a slow decrease after the initial rise, the telehealth visit rate still increased by 97.5% (2.35 visits per person-year; 95% CI 86.0%-109.7%) in July-December 2021 compared to the prepandemic period, comprising approximately 42% of outpatient visits (5.63 visits per person-year; Table 3). Combined telehealth and outpatient visits increased by 5.5% (95% CI 0.7%-10.6%) and combined in-person visits decreased by 11.9% (95% CI –16.0% to –7.7%) in July-December 2021 compared to prepandemic levels (Table 3).

**Table 1:** Enrollment in person-years by demographic characteristics during 2017-2021 among all members.

Characteristics			Enrollment (person-years), n (%)								
			2017 (n=10,881,765)		2018 (n=11,240,470)		2019 (n=11,344,837)		2020 (n=11,489,491)		2021 (n=11,487,605)
**Age group (years)**											
	0-5	689,315 (6.3)		705,836 (6.3)		705,341 (6.2)		702,643 (6.1)		683,424 (6)	
	6-17	1,575,104 (14.5)		1,601,089 (14.2)		1,602,148 (14.1)		1,607,990 (14.0)		1,590,119 (13.8)	
	18-44	4,015,752 (36.9)		4,183,223 (37.2)		4,236,994 (37.4)		4,311,066 (37.5)		4,311,670 (37.5)	
	45-64	2,981,324 (27.4)		3,064,043 (27.3)		3,058,884 (27)		3,073,703 (26.8)		3,053,622 (26.6)	
	65+	1,620,270 (14.9)		1,686,279 (15)		1,741,470 (15.4)		1,794,089 (15.6)		1,848,770 (16.1)	
**Sex**											
	Female	5,650,298 (51.9)		5,836,729 (51.9)		5,890,756 (51.9)		5,967,580 (51.9)		5,972,430 (52)	
	Male	5,230,911 (48.1)		5,403,074 (48.1)		5,453,024 (48.1)		5,520,266 (48.1)		5,512,942 (48)	
	Unknown	556 (0.01)		667 (0.01)		1056 (0.01)		1645 (0.01)		2233 (0.02)	
**Race or ethnicity**											
	Hispanic	2,756,704 (25.3)		2,853,922 (25.4)		2,909,586 (25.7)		2,957,211 (25.7)		2,978,880 (25.9)	
	Non-Hispanic Asian	1,294,988 (11.9)		1,366,584 (12.2)		1,408,848 (12.4)		1,439,991 (12.5)		1,453,505 (12.7)	
	Non-Hispanic Black	742,060 (6.8)		759,581 (6.8)		762,394 (6.7)		767,604 (6.7)		767,941 (6.7)	
	Non-Hispanic multiple or other	473,214 (4.4)		492,853 (4.4)		501,299 (4.4)		508,699 (4.4)		512,073 (4.5)	
	Non-Hispanic White	4,756,019 (43.7)		4,832,183 (43.0)		4,774,036 (42.1)		4,704,680 (41.0)		4,582,321 (39.9)	
	Unknown	858,780 (7.9)		935,347 (8.3)		988,673 (8.7)		1,111,306 (9.7)		1,192,884 (10.4)	

**Table 2:** Visit rates before and during the early COVID-19 pandemic in 2020 and adjusted percent change from prepandemic among all members.

			Visit rate in 2020 (per person-year)									Adjusted percent change (95% CI)^a^				
			Jan-Feb		Apr-Jun		Jul-Sep		Oct-Dec		Apr-Jun			Jul-Sep		Oct-Dec
Overall			7.82		5.72		7.16		7.72		–*26.4* (*–36.3 to –15.1*)			–*6.4* (*–13.2 to 0.9*)		–0.4 (–6.3 to 5.9)
**Setting**																
	IP^b^	.108		.080		.098		.099		–*24.9* (*-33.3 to –15.3*)			–*7.2* (*–10.5 to –3.7*)		–*5.3* (*–8.3 to –2.2*)	
	ED^c^	.288		.185		.228		.227		–*35.5* (*–43.7 to –26.2*)			–*19.5* (*–22.2 to –16.6*)		–*19.2* (*–22.7 to –15.5*)	
	OP^d^	6.42		2.28		3.75		4.42		–*64.2* (*–71.9 to –54.3*)			–*40.3* (*–45.5 to –34.7*)		–*30.5* (*–34.4 to –26.4*)	
	TH^e^	1.01		3.17		3.08		2.98		*218.3* (*186.4 to 253.6*)			*218.1* (*187.8 to 251.5*)		*192.6* (*168.6 to 218.6*)	
	OP+TH	7.43		5.45		6.83		7.40		–*26.1* (*–36.1 to –14.6*)			–5.9 (–13.0 to 1.8)		0.4 (–5.9 to 7.1)	
	In-person^f^	6.81		2.55		4.08		4.74		–*62.4* (*–70.2 to –52.5*)			–*38.9* (*–44.0 to –33.5*)		–*29.7* (*–33.4 to –25.7*)	

^a^Italic formatting indicates if *P*<.05.

^b^IP: inpatient.

^c^ED: emergency department.

^d^OP: outpatient.

^e^TH: telehealth.

^f^In-person: IP+ED+OP.

**Table 3:** Visit rates during 2017-2019 and the late COVID-19 pandemic in 2021 and adjusted percent change from prepandemic among all members.

		Visit rate (per person-year)				Adjusted rate ratio (95% CI)^a^	Adjusted percent change (95% CI)^a^
		Jul-Dec 2017	Jul-Dec 2018	Jul-Dec 2019	Jul-Dec 2021		
Overall		6.89	7.11	7.49	8.36	*1.05* (*1.01 to 1.10*)	*5.1* (*0.6 to 9.9*)
**Setting**							
	IP^b^	0.105	0.104	0.105	0.103	0.98 (0.96 to 1.00)	–1.9 (–4.0 to 0.2)
	ED^c^	0.259	0.262	0.271	0.275	0.98 (0.95 to 1.01)	–2.0 (–5.0 to 1.0)
	OP^d^	5.91	6.05	6.24	5.63	*0.87* (*0.83 to 0.92*)	–*12.5* (*–16.8 to –8.0*)
	TH^e^	0.625	0.694	0.865	2.35	*1.97* (*1.86 to 2.10*)	*97.5* (*86.0 to 109.7*)
	OP+TH	6.53	6.74	7.11	7.98	*1.05* (*1.01 to 1.11*)	*5.5* (*0.7 to 10.6*)
	In-person^f^	6.27	6.42	6.62	6.01	*0.88* (*0.84 to 0.92*)	–*11.9* (*–16.0 to –7.7*)

^a^Italic formatting indicates if *P*<.05.

^b^IP: inpatient.

^c^ED: emergency department.

^d^OP: outpatient.

^e^TH: telehealth.

^f^In-person: IP+ED+OP.

Enrolled person-years during 2017-2021 ranged from 8.8 to 9.0 million after excluding members with evidence of COVID-19. Demographic characteristics of members without COVID-19 remained similar compared to all members (Table S1 in Multimedia Appendix 2). The trends of overall monthly visit rates among members without COVID-19 and all members were similar (Figure 1). The overall visit rate among members without COVID-19 was lower than that of all members, and the difference increased during the pandemic. The overall visit rate among members without COVID-19 decreased by 5.5% (95% CI –10.3% to –0.5%) in October-December 2020 from 7.37 visits per person-year in January-February 2020 to 6.85 visits per person-year in October-December 2020; inpatient and ED visit rates decreased by 15.6% (95% CI –21.1% to –9.8%) and 28.6% (95% CI –35.0% to –21.5%) from 0.107 and 0.274 visits per person-year in January-February 2020 to 0.087 and 0.189 visits per person-year in October-December 2020, respectively (Table S2 in Multimedia Appendix 2). In July-December 2021, the overall visit rate among members without COVID-19 returned to prepandemic levels, while the inpatient and ED visit rates were still 7.5% (95% CI –9.6% to –5.3%) and 8.0% (95% CI –11.0% to –4.9%) lower, reaching 0.095 and 0.241 visits per person-year, respectively (Table S3 in Multimedia Appendix 2).

There were around 4.5 million enrolled person-years in KPSC each year during the study period. The use trends overall and by visit type at KPSC were similar to those of the entire study population during 2017-2021 (Figures S1 and S2 in Multimedia Appendix 3). The visit rates from January 2022 to June 2023 remained stable, continuing at the late 2021 levels. The adjusted percent changes of visit rates in July-December 2022 were consistent with the estimates from the main analysis (Table S1 in Multimedia Appendix 3). The overall, inpatient, ED, and combined telehealth and outpatient visit rates among all members returned to prepandemic levels during July-December 2022. The telehealth visit rate in July-December 2022 increased by 92.3% (95% CI 60.3%-130.7%) compared to the prepandemic period. Among members without COVID-19, the inpatient and ED visit rates during July-December 2022 were still 11.1% (95% CI –14.0% to –8.0%) and 10.2% (95% CI –15.3% to –4.9%) lower than prepandemic levels, respectively.

## Discussion

### Principal Findings

In this large, retrospective cohort study conducted across 8 integrated health care systems with more than 11 million demographically and geographically diverse members, the reductions observed in the early pandemic period persisted into late 2020 for in-person visits, but overall use and counting all types of visits tended to equilibrate because of dramatically increased use of telehealth. Furthermore, our study showed the longer-term impact of the COVID-19 pandemic on health care utilization, especially after COVID-19 restrictions were relaxed. Specifically, during late 2021, inpatient and ED visit rates returned to prepandemic levels and overall use exceeded prepandemic levels. The trends observed in the KPSC data closely mirrored those of the entire study population. The trends during the extended period from January 2022 to June 2023 were stable and appeared to be a continuation of the levels observed at the end of 2021. KPSC contributed over one-third of the data.

Telehealth care was promoted and supported through reimbursement during the COVID-19 pandemic [18], which partially resulted in a surge in telehealth visits during the COVID-19 pandemic. Although the rates decreased after an initial rise, they remained doubled in late 2021 compared to prepandemic levels. Telehealth visits increased slowly before the COVID-19 pandemic due to individual and organizational barriers, which have been discussed in previous studies [19]. The COVID-19 pandemic provided an opportunity for health care systems, providers, and patients to adopt this new approach for health care delivery [20,21]. Furthermore, studies have shown that minority and low-income groups could benefit significantly from the increased accessibility of telehealth care by eliminating barriers such as transportation costs and time constraints [22,23]. Despite the fact that telehealth may not be appropriate for certain medical conditions (eg, physical examinations, medical procedures, and emergent treatments) [24] and access to in-person care improved later in the COVID-19 pandemic, telehealth care remained a mainstay of health care delivery, comprising approximately 42% (5.63 visits per person-year) of outpatient visits in late 2021 (2.35 telehealth visits per person-year). The postpandemic use of telehealth is likely to continue growing and evolving to improve patient outcomes, access to care, and convenience of care [25]. These historical trends can help inform projections for the use of telehealth and in-person modalities for care.

COVID-19–related care added substantially to the burden on the health care system [26]. During the early stages of the COVID-19 pandemic, there was competition between the resources allocated to COVID-19–related care and those directed toward non–COVID-19–related medical needs [2]. Both types of care required significant resources, potentially straining the health care system’s capacity. Excluding members with COVID-19 and all their use during the study period did not significantly change the direction of health care utilization trends in this population; however, the visit rates decreased more among members without evidence of COVID-19 during the pandemic, especially in the inpatient and ED settings. In the later pandemic period, inpatient and ED visits among members without evidence of COVID-19 decreased by 7.5% and 8% (from 0.105 and 0.258 visits per person-year in prepandemic to 0.095 and 0.241 visits per person-year in later pandemic), respectively, and these rates did not return to prepandemic levels. While there were differences in baseline use between all members and the members without COVID-19 possibly due to differences in health status and care-seeking behavior, both DiD and ITS analyses accounted for the baseline use within each group. As we adjust to the post–COVID-19 pandemic phase, it is important to investigate the reasons behind this phenomenon and prioritize efforts that address the possible unmet needs of those patients with non–COVID-19 illness.

Knowledge of health care utilization is also important for researchers who conduct studies using observational data during the pandemic period. For example, vaccine safety studies commonly identified outcomes from in-person encounters before the COVID-19 pandemic [12]. Since telehealth use remained high during the pandemic, which contributed to the delivery of health care beyond in-person visits, telehealth visits could be an important data source for identifying adverse events in vaccine safety studies during or after the pandemic period. In addition, with the changes in use over time, secular confounding needs to be considered when designing a study involving the pandemic period and selecting appropriate unbiased comparison groups.

There are several statistical considerations for these analyses. First, this study conducted analyses at the population level. We showed that the demographic characteristics of members enrolled at VSD sites were stable over the study period. Both DiD analysis and ITS analyses compared health care utilization before and during the COVID-19 pandemic among a similar population. Hence, patient-level adjustment was not critical. Second, unmeasured secular confounders (eg, changes in care practice unrelated to COVID-19 and changes in underlying health conditions of the study population) were adjusted for through either the DiD approach or a prepandemic trend estimated from 2017 to 2019 data. Although the latter required an assumption that the trend would have continued through 2020-2021 if there was no COVID-19 pandemic, the assumption likely held, given the moderate length of the study period. The study also had some limitations. The study aimed to assess the overall impact of the COVID-19 pandemic on health care utilization, including factors such as the population’s fear of exposure in health care settings, policy changes in physical distancing, surges of new COVID-19 cases, and COVID-19 vaccinations. To assess the individual impact of each factor would require proper measurement of these factors and a different study design and analysis. In addition, the study did not assess the impact of the COVID-19 pandemic on subgroups. The impact could differ by age, race or ethnicity, socioeconomic status, and health conditions, as has been shown in previous studies [22,27]. Finally, we included persons enrolled in health plans; therefore, our findings may not be generalizable to the uninsured population.

### Conclusions

During the late COVID-19 pandemic period, overall use of health care services exceeded prepandemic levels as telehealth services became a mainstay of the health care system. Inpatient and ED visits returned to prepandemic levels, although they remained low among members without evidence of COVID-19. Our findings provide valuable information for planning longer term strategic resource allocation for patient care in the postpandemic period and for designing observational studies involving the pandemic period.

## Appendix

Multimedia Appendix 1 Illustration of the periods compared in the difference-in-difference analysis and interrupted time series analysis.

Multimedia Appendix 2 Health care utilization among members without COVID-19.

Multimedia Appendix 3 Health care utilization in Kaiser Permanente Southern California during 2017-2023.

## Abbreviations

DiD: difference-in-difference
ED: emergency department
ITS: interrupted time series
KPSC: Kaiser Permanente Southern California
RR: rate ratio
RRR: ratio of rate ratio
VSD: Vaccine Safety Datalink

## Data Availability

The data sets generated during this study are not available for public access. Guidelines on how to access Vaccine Safety Datalink data through a sharing program administered by the National Center for Health Statistics Research Data Center are provided on the Vaccine Safety Datalink website and are subject to change.
