# Supplementary material for: Impact of the COVID-19 Pandemic on Health Care Utilization in the Vaccine Safety Datalink: Retrospective Cohort Study
Source: JMIR Public Health Surveill. 2024 Jan 23;10:e48159. doi: 10.2196/48159 (PMC10807656; doi:10.2196/48159)

Figure S1. Illustration of the periods compared in the difference-in-difference analysis and interrupted time series analysis.


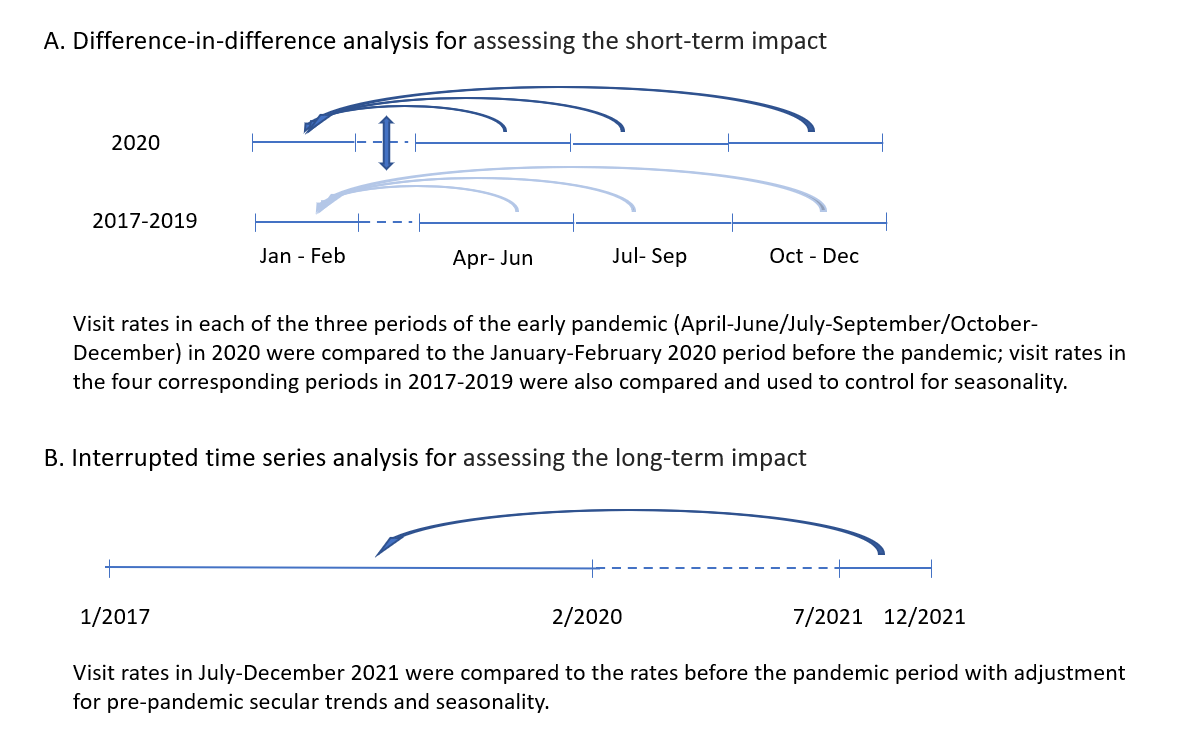

Supplement: Multimedia Appendix 1 [file publichealth_v10i1e48159_app1.doc]
