# Supplementary material for: Impact of the COVID-19 Pandemic on Health Care Utilization in the Vaccine Safety Datalink: Retrospective Cohort Study
Source: JMIR Public Health Surveill. 2024 Jan 23;10:e48159. doi: 10.2196/48159 (PMC10807656; doi:10.2196/48159)
Supplement: Multimedia Appendix 2 [file publichealth_v10i1e48159_app2.doc]

Table S1. Enrollment in person-years by demographic characteristics during 2017-2021 among members without COVID-19.

|  | Enrollment (person-years) | | | | |
| --- | --- | --- | --- | --- | --- |
|  | 2017 | 2018 | 2019 | 2020 | 2021 |
| Overall | 8,820,187 | 9,022,092 | 8,990,776 | 8,965,318 | 8,887,869 |
| Age (years) |  |  |  |  |  |
| 0-5 | 569,640 (6.5%) | 580,418 (6.4%) | 576,307 (6.4%) | 568,287 (6.3%) | 547,537 (6.2%) |
| 6-17 | 1,280,558 (14.5%) | 1,294,063 (14.3%) | 1,286,597 (14.3%) | 1,281,472 (14.3%) | 1,260,465 (14.2%) |
| 18-44 | 3,195,570 (36.2%) | 3,288,719 (36.5%) | 3,274,583 (36.4%) | 3,260,804 (36.4%) | 3,225,555 (36.3%) |
| 45-64 | 2,398,520 (27.2%) | 2,439,401 (27.0%) | 2,400,566 (26.7%) | 2,371,719 (26.5%) | 2,329,492 (26.2%) |
| 65+ | 1,375,899 (15.6%) | 1,419,491 (15.7%) | 1,452,723 (16.2%) | 1,483,036 (16.5%) | 1,524,820 (17.2%) |
| Sex |  |  |  |  |  |
| Female | 4,530,062 (51.4%) | 4,632,686 (51.3%) | 4,615,078 (51.3%) | 4,600,751 (51.3%) | 4,563,575 (51.4%) |
| Male | 4,289,676 (48.6%) | 4,388,875 (48.7%) | 4,374,885 (48.7%) | 4,363,273 (48.7%) | 4,322,561 (48.6%) |
| Unknown | 450 (0.01%) | 531 (0.01%) | 811 (0.01%) | 1,294 (0.01%) | 1,733 (0.02%) |
| Race/Ethnicity |  |  |  |  |  |
| Hispanic | 2,151,562 (24.4%) | 2,198,402 (24.4%) | 2,203,719 (24.5%) | 2,189,894 (24.4%) | 2,188,673 (24.6%) |
| Non-Hispanic Asian | 1,113,400 (12.6%) | 1,168,348 (12.9%) | 1,195,661 (13.3%) | 1,211,242 (13.5%) | 1,216,022 (13.7%) |
| Non-Hispanic Black | 606,267 (6.9%) | 612,569 (6.8%) | 606,771 (6.7%) | 600,284 (6.7%) | 593,743 (6.9%) |
| Non-Hispanic Multiple/Other | 386,049 (4.4%) | 398,176 (4.4%) | 399,817 (4.4%) | 398,496 (4.4%) | 396,573 (4.5%) |
| Non-Hispanic White | 3,801,899 (43.1%) | 3,826,177 (42.4%) | 3,735,012 (41.5%) | 3,629,081 (40.5%) | 3,500,341 (39.4%) |
| Unknown | 761,011 (8.6%) | 818,420 (9.1%) | 849,795 (9.5%) | 936,321 (10.4%) | 992,516 (11.2%) |

Table S2. Visit rates before and during the early COVID-19 pandemic in 2020 and adjusted percent change from pre-pandemic among members without COVID-19.

|  | Visit rate in 2020 (per person-year) | | | | Adjusted percent change (95% CI)^a^ | | |
| --- | --- | --- | --- | --- | --- | --- | --- |
|  | January-February | April-June | July-September | October-December | April-June | July-September | October-December |
| Overall | 7.37 | 5.24 | 6.52 | 6.85 | **-28.4 (-37.9, -17.5)** | **-9.4 (-16.1, -2.1)** | **-5.5 (-10.3, -0.5)** |
| Setting^b^ |  |  |  |  |  |  |  |
| IP | .107 | .075 | .091 | .087 | **-28.4 (-36.8, -19.0)** | **-12.2 (-15.9, -8.3)** | **-15.6 (-21.1, -9.8)** |
| ED | .274 | .168 | .203 | .189 | **-37.9 (-45.8, -28.9)** | **-23.9 (-26.6, -21.1)** | **-28.6 (-35.0, -21.5)** |
| OP | 6.03 | 2.06 | 3.40 | 3.90 | **-65.5 (-73.0, -56.0)** | **-42.4 (-47.6, -36.7)** | **-34.0 (-37.6, -30.2)** |
| TH | .962 | 2.93 | 2.83 | 2.66 | **207.9 (177.3, 241.9)** | **206.4 (178.8, 236.6)** | **176.4 (157.2, 197.1)** |
| OP+TH | 6.99 | 4.99 | 6.23 | 6.57 | **-28.1 (-37.7, -16.9)** | **-8.8 (-15.8, -1.1)** | -4.5 ( -9.7, 1.1) |
| In-person | 6.41 | 2.31 | 3.69 | 4.18 | **-63.7 (-71.3, -54.2)** | **-41.1 (-46.2, -35.6)** | **-33.5 (-36.9, -29.9)** |

^a^ Bold if *P* <.05

^b^ IP=Inpatient, ED=Emergency department, OP=Outpatient, TH=Telehealth, In-person=IP+ED+OP

Table S3. Visit rates during 2017-2019 and the late COVID-19 pandemic in 2021 and adjusted percent change from pre-pandemic among members without COVID-19.

|  | Visit rate (per person-year) | | | | Adjusted rate ratio  (95% CI)^a^ | Adjusted percent change (95% CI)^a^ |
| --- | --- | --- | --- | --- | --- | --- |
|  | 2017  July-December | 2018  July-December | 2019  July-December | 2021  July-December |  |  |
| Overall | 6.65 | 6.82 | 7.08 | 7.64 | 1.03 (0.99, 1.08) | 3.2 (-1.3, 7.8) |
| Setting^b^ |  |  |  |  |  |  |
| IP | .106 | .105 | .105 | .095 | **0.93 (0.90, 0.95)** | **-7.5 (-9.6, -5.3)** |
| ED | .252 | .253 | .258 | .241 | **0.92 (0.89, 0.95)** | **-8.0 (-11.0, -4.9)** |
| OP | 5.69 | 5.79 | 5.89 | 5.14 | **0.86 (0.82, 0.90)** | **-13.9 (-18.1, -9.5)** |
| TH | .608 | .672 | .830 | 2.16 | **1.91 (1.80, 2.03)** | **91.5 (80.4, 103.2)** |
| OP+TH | 6.30 | 6.46 | 6.72 | 7.30 | 1.04 (0.99, 1.09) | 3.7 (-1.0, 8.7) |
| In-person | 6.05 | 6.15 | 6.25 | 5.48 | **0.86 (0.82, 0.91)** | **-13.6 (-17.5, -9.4)** |

^a^ Bold if *P* <.05

^b^ IP=Inpatient, ED=Emergency department, OP=Outpatient, TH=Telehealth, In-person=IP+ED+OP
