# Supplementary material for: Impact of the COVID-19 Pandemic on Health Care Utilization in the Vaccine Safety Datalink: Retrospective Cohort Study
Source: JMIR Public Health Surveill. 2024 Jan 23;10:e48159. doi: 10.2196/48159 (PMC10807656; doi:10.2196/48159)
Supplement: Multimedia Appendix 3 [file publichealth_v10i1e48159_app3.doc]

Multimedia Appendix 3.

Figure S1. Monthly overall visit rate among all members and members without COVID-19, 2017-2023, Kaiser Permanente Southern California.


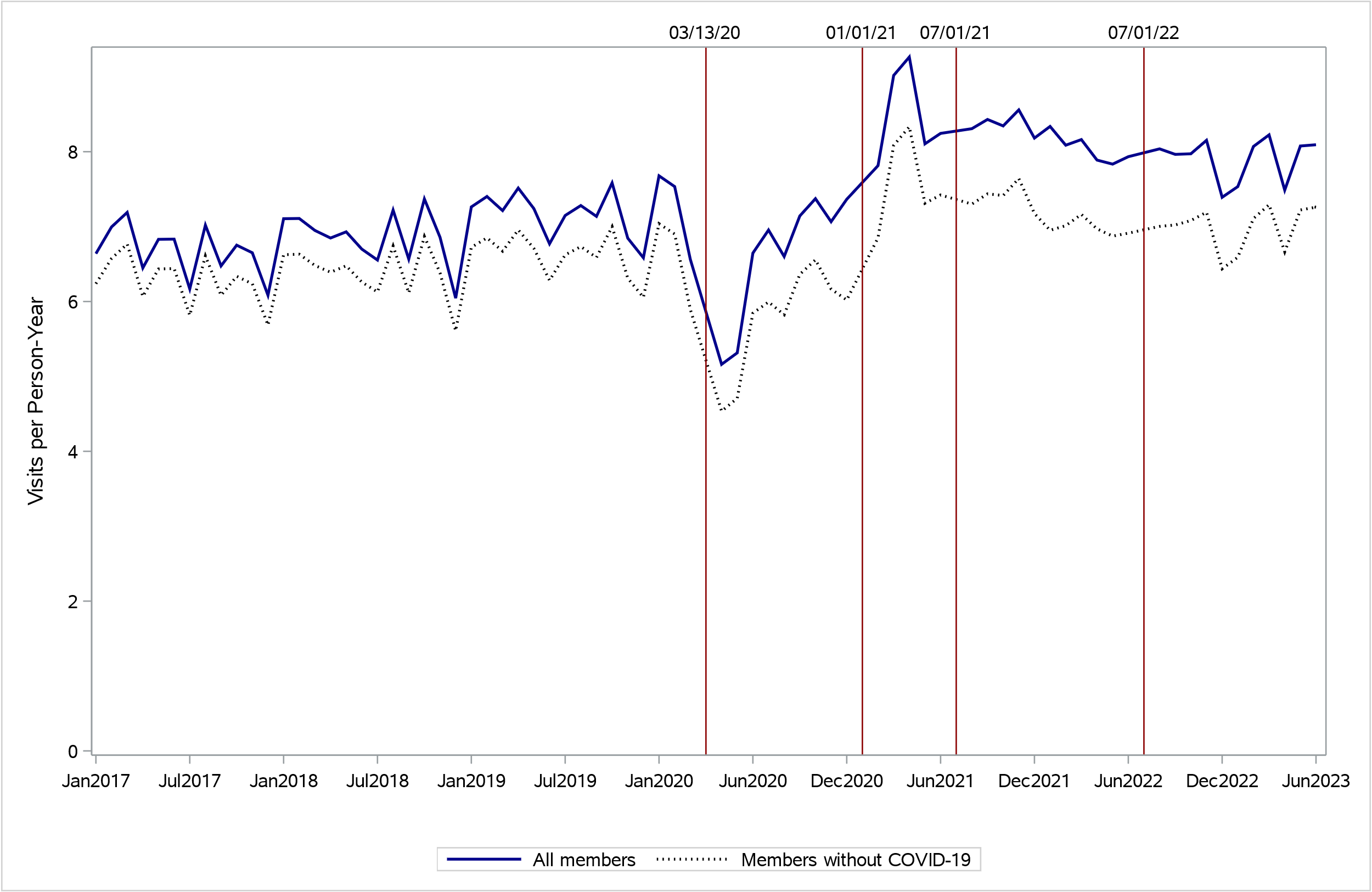


Definition of periods: pre-pandemic period: January 1, 2017-March 13, 2020; early pandemic period: April-December 2020; later pandemic period: July-December 2021; extended pandemic period corresponding to July-December 2021: July-December 2022.

Figure S2. Monthly visit rate by care setting among all members, 2017-2023, Kaiser Permanente Southern California.
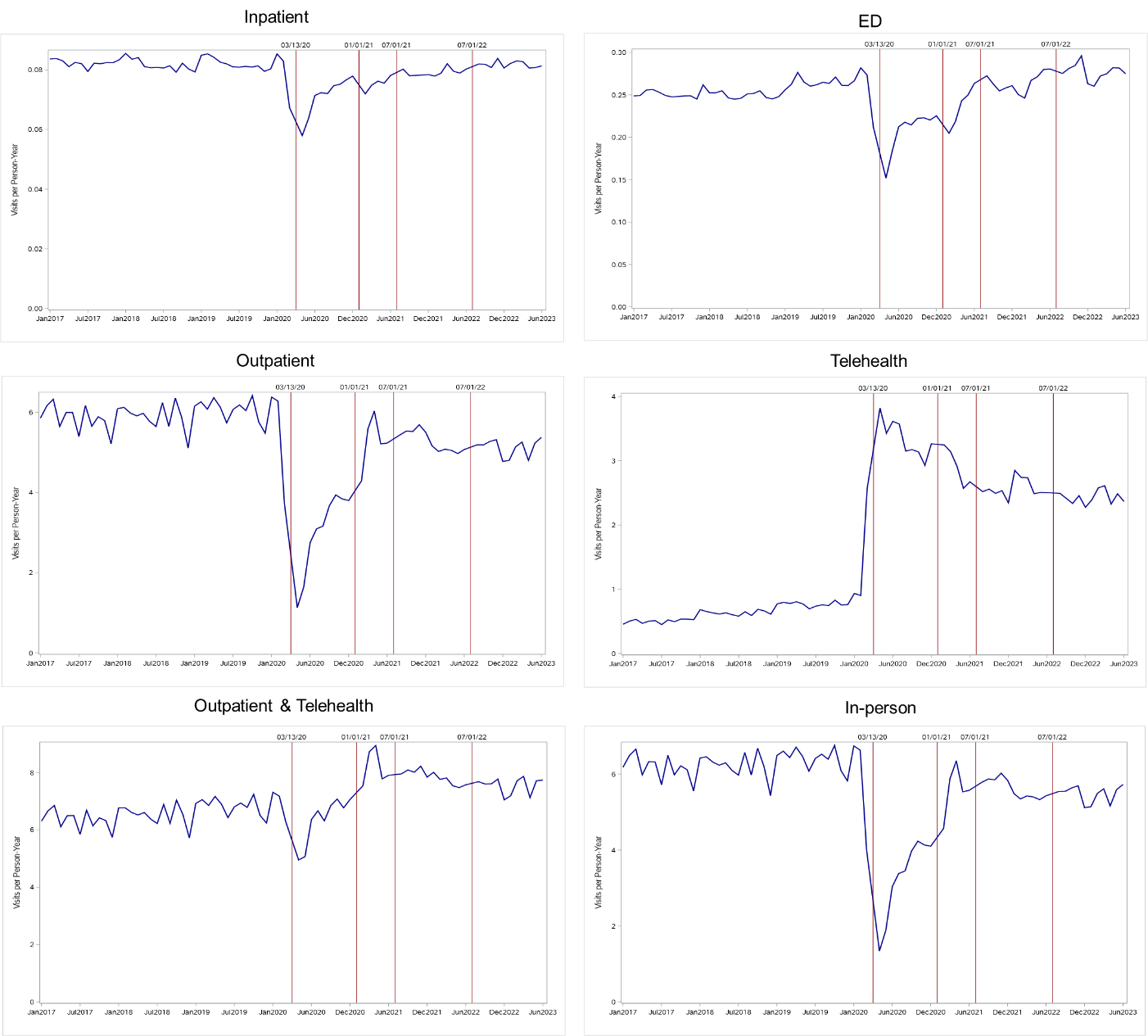


Definition of periods: pre-pandemic period: January 1, 2017-March 13, 2020; early pandemic period: April-December 2020; later pandemic period: July-December 2021; extended pandemic period corresponding to July-December 2021: July-December 2022.

Table S1. Visit rates during 2017-2019 and 2021-2022 and adjusted percent change from pre-pandemic among all members and members without COVID-19 at Kaiser Permanente Southern California.

|  | 2017 | 2018 | 2019 | 2021 | 2022 | Adjusted percent change (95% CI)  2021 vs. Pre-pandemic^a^ | Adjusted percent change (95% CI)  2022 vs. Pre-pandemic^a^ |
| --- | --- | --- | --- | --- | --- | --- | --- |
|  | July - December | July - December | July - December | July - December | July - December |
| All members | | | | | | | |
| Overall | 6.52 | 6.77 | 7.10 | 8.25 | 7.77 | **9.0 (4.1, 14.0)** | -1.0 (-6.3, 4.7) |
| Setting^b^ |  |  |  |  |  |  |  |
| IP | .082 | .080 | .081 | .079 | .081 | **-2.3 ( -4.3, -0.3)** | 0.9 (-1.5, 3.4) |
| ED | .250 | .250 | .265 | .264 | .280 | **-5.4 ( -9.2, -1.4)** | -2.7 (-7.3, 2.3) |
| OP | 5.68 | 5.81 | 5.99 | 5.43 | 5.05 | **-11.8 (-16.3, -7.1)** | **-19.5 (-24.4, -14.3)** |
| TH | .510 | .630 | .764 | 2.47 | 2.36 | **114.4 (102.2, 127.4)** | **92.3 (60.3, 130.7)** |
| OP+TH | 6.19 | 6.44 | 6.75 | 7.90 | 7.41 | **9.7 (4.6, 15.0)** | -0.9 (-6.5, 5.0) |
| IP+ED+OP | 6.01 | 6.14 | 6.33 | 5.77 | 5.41 | **-11.4 (-15.7, -6.9)** | **-18.5 (-23.3, -13.5)** |
| Members without COVID-19 | | | | | | | |
| Overall | 6.13 | 6.31 | 6.55 | 7.30 | 6.80 | **6.2 (1.3, 11.4)** | -3.6 (-9.0, 2.1) |
| Setting^b^ |  |  |  |  |  |  |  |
| IP | .082 | .080 | .079 | .068 | .068 | **-11.9 (-14.3, -9.4)** | **-11.1 (-14.0, -8.0)** |
| ED | .240 | .237 | .246 | .216 | .229 | **-13.8 (-17.9, -9.6)** | **-10.2 (-15.3, -4.9)** |
| OP | 5.34 | 5.42 | 5.54 | 4.90 | 4.53 | **-12.7 (-17.2, -7.8)** | **-20.2 (-25.2, -14.8)** |
| TH | .469 | .575 | .686 | 2.11 | 1.98 | **107.7 (95.3, 120.9)** | **87.3 (55.1, 126.2)** |
| OP+TH | 5.80 | 5.99 | 6.23 | 7.01 | 6.51 | **7.2 (2.0, 12.6)** | -3.3 (-9.0, 2.7) |
| IP+ED+OP | 5.66 | 5.73 | 5.87 | 5.19 | 4.83 | **-12.7 (-17.1, -8.1)** | **-19.6 (-24.4, -14.5)** |

^a^ Bold if *P* <.05

^b^ IP=Inpatient, ED=Emergency department, OP=Outpatient, TH=Telehealth, In-person=IP+ED+OP
